# A Novel Engineering Cell Therapy Platform Mimicking the Immune Thrombocytopenia‐Derived Platelets to Inhibit Cytokine Storm in Hemophagocytic Lymphohistiocytosis

**DOI:** 10.1002/advs.202404571

**Published:** 2024-09-11

**Authors:** Zhenyu Liu, Ying Du, Tong Zhou, Ting Qin, Yining Yuan, Weilu Xu, MengKun Fang, Xuemei Wang, Bing Chen, Peipei Xu

**Affiliations:** ^1^ Department of Hematology Nanjing Drum Tower Hospital Affiliated Hospital of Medical School Nanjing University Nanjing 210008 China; ^2^ Department of Hematology Nanjing Drum Tower Hospital Clinical College of Nanjing Medical University Nanjing 210008 China; ^3^ School of Biological Science & Medical Engineering Southeast University Nanjing 210096 China

**Keywords:** cytokine storm, etoposide, hemophagocytic lymphohistiocytosis, living therapeutics platform, platelets

## Abstract

Hemophagocytic lymphohistiocytosis (HLH) is a common and highly fatal hyperinflammatory syndrome characterized by the aberrant activation of macrophages. To date, there is a lack of targeted therapies for HLH. It is validated that macrophages in HLH efficiently phagocytose anti‐CD41‐platelets (anti‐CD41‐PLTs) from immune thrombocytopenia (ITP) patients in previous research. Hence, the pathological mechanisms of ITP are mimicked and anti‐CD41‐PLTs are utilized to load the macrophage‐toxic drug VP16 to construct macrophage‐targetable engineered platelets anti‐CD41‐PLT‐VP16, which is a novel targeted therapy against HLH. Both in vitro and in vivo studies demonstrate that anti‐CD41‐PLT‐VP16 has excellent targeting and pro‐macrophage apoptotic effects. In HLH model mice, anti‐CD41‐PLT‐VP16 prevents hemophagocytosis and inhibits the cytokine storm. Mechanistic studies reveal that anti‐CD41‐PLT‐VP16 increases the cytotoxicity of VP16, facilitating precise intervention in macrophages. Furthermore, it operates as a strategic “besieger” in diminishing hyperinflammation syndrome, which can indirectly prevent the abnormal activation of T cells and NK cells and reduce the Ab‐dependent cell‐mediated cytotoxicity effect. The first platelet‐based clinical trial is ongoing. The results show that after treatment with anti‐CD41‐PLT‐VP16, HLH patients have a threefold increase in the overall response rate compared to patients receiving conventional chemotherapy. In conclusion, anti‐CD41‐PLT‐VP16 provides a general insight into hyperinflammation syndrome and offers a novel clinical therapeutic strategy for HLH.

## Graphical abstract

Inspired by the characteristics of platelets in immune thrombocytopenia that are easily phagocytosed by macrophages, this study designs and constructs anti‐CD41‐PLT‐VP16 for targeting abnormally activated macrophages in hemophagocytic lymphohistiocytosis (HLH). Both in vitro and in vivo studies show that anti‐CD41‐PLT‐VP16 has a better therapeutic effect on HLH.

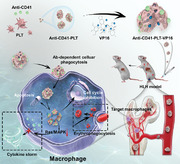

## 1 Introduction

Hemophagocytic lymphohistiocytosis (HLH) is a hyperinflammatory syndrome that can be induced by numerous diseases, including infection, malignancy, and autoimmune disease. HLH is characterized by severe symptoms and a high mortality rate.^[^ ^1^, ^2^, ^3^ ^]^ Activated macrophages play a crucial role in HLH by engulfing many blood cells.^[^ ^4^, ^5^, ^6^, ^7^, ^8^, ^9^ ^]^ During the pathogenic process, macrophages also release high levels of cytokines such as IFN‐γ, TNF‐α, and IL‐6, leading to a cytokine storm.^[^ ^10^, ^11^, ^12^ ^]^ In addition, aberrant macrophages can promote the activation of cytotoxic T cells and natural killer (NK) cells, further exacerbating cytokine release. Ultimately, patients often die due to multiple organ dysfunction syndrome (MODS). Therefore, inhibiting abnormally activated macrophages is a priority in treating HLH^[^ ^13^ ^]^ Etoposide (VP16) is a drug with strong effects on macrophages and is the first‐line drug for HLH treatment, but its efficacy is still not ideal. Thus, effective intervention strategies for HLH are lacking.

Platelet (PLT)–based cellular therapies have been shown to be uniquely superior and promising.^[^ ^14^, ^15^, ^16^ ^]^ Interestingly, our previous studies have revealed that the phagocytosis of PLTs from patients with primary immune thrombocytopenia (ITP), anti‐CD41‐PLTs, by macrophages is 15–35 times more efficient than that of normal PLTs.^[^ ^17^, ^18^ ^]^ Thus, anti‐CD41‐PLTs can be efficiently phagocytosed by abnormally activated macrophages in HLH and reduce macrophage activity by acting as a “besieger” through the antibody‐dependent cellular phagocytosis (ADCP), thus preventing secondary Ab‐dependent cell‐mediated cytotoxicity (ADCC) effects.^[^ ^15^, ^19^, ^20^ ^]^

**Scheme 1:**
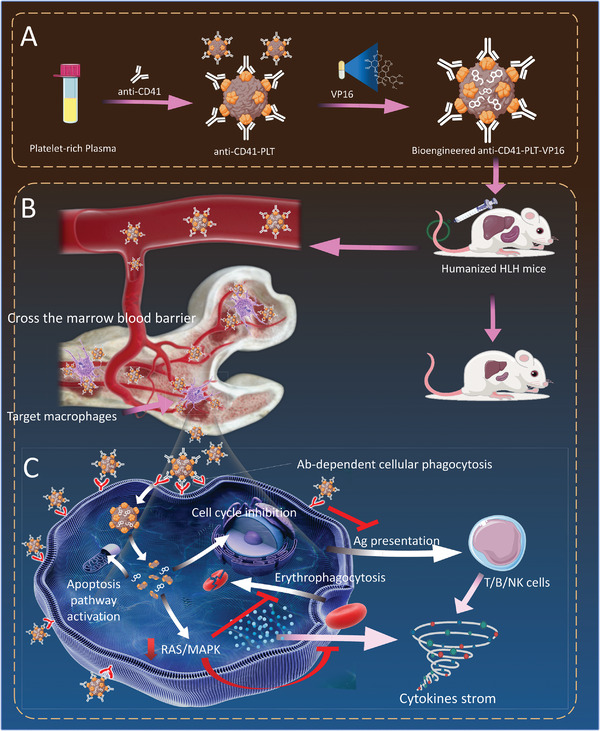
Schematic illustration of the anti‐CD41‐PLT‐VP16 process and relevant therapeutic effect on HLH mice. Anti‐CD41‐PLT‐VP16 blocks phagocytosis reduces cytokine storms, and indirectly inhibits the function of other hyperinflammation‐associated immune cells in the form of “besieger”.

Inspired by the fact that platelets from ITP patients are more readily phagocytosed by macrophages, and synthesizing the physiological benefits of PLTs as drug carriers, we screened some patients with primary ITP whose platelets were modified by anti‐CD41 mAb and verified the ability of anti‐CD41‐PLT to target macrophages.^[^ ^21^, ^22^ ^]^ Then the platelets products were used to construct anti‐CD41‐PLT, which mimicked platelets from ITP patients, and piggybacked VP16 with it, resulting in a platelet‐based in vivo therapeutic platform, anti‐CD41‐PLT‐VP16, and used it to treat HLH. The PLTs modified with anti‐CD41a to precisely and effectively allow VP16 to target abnormally activated macrophages, thus realizing long‐lasting and precise treatment of HLH.^[^ ^15^, ^23^, ^24^ ^]^ After successfully constructing anti‐CD41‐PLT‐VP16, we measured its ability to target and inhibit macrophage activity. This study investigated the inhibitory effect and safety of anti‐CD41‐PLT‐VP16 on overactive macrophages of HLH, both in vitro and in vivo (**Scheme** **1**). In addition, this study has preliminarily explored the mechanism by which anti‐CD41‐PLT‐VP16 alleviates HLH by decreasing macrophage activity and inhibiting hyperinflammatory responses. The first clinical trial for HLH patients based on anti‐CD41‐PLT‐VP16 in Asia is ongoing, resulting in preliminary validation of its effectiveness. The initiation of clinical trials paves the way for exploring the application value of the mAb‐opsonized PLT living therapeutics platform.

## 2 Results

### 2.1 Anti‐CD41‐PLT‐VP16 Has a High Drug‐Loading Rate and Can Control the Release of Drugs

**Figure 1:**
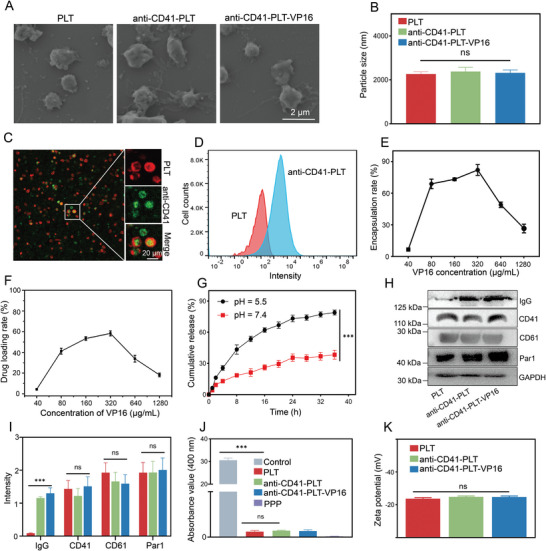
Characterization and properties of anti‐CD41‐PLT‐VP16. A) PLT morphology was assessed by electron microscopy. Bar = 2 µm. B) DLS was used to measure the size of the PLTs in each group. C) CLSM image showing the colocalization of anti‐CD41‐PLT‐VP16 components. (PLT: red, anti‐CD41: green, bar = 20 µm) D) FCM verified the labeling efficiency of anti‐CD41 on PLTs. E) HPLC was used to determine the encapsulation rate of anti‐CD41‐PLT‐VP16 at multiple concentrations. VP16 at 320 µg mL^−1^ resulted in the optimal encapsulation rate, so this concentration was used for constructing anti‐CD41‐PLT‐VP16 in subsequent experiments. F) HPLC was used to detect the loading rate of multiple concentrations of anti‐CD41‐PLT‐VP16. G) HPLC was used to detect the drug release rate from pharmacophores in different pH environments; pH 7.4 was used to simulate in vivo circulation, and pH 5.5 was used to simulate macrophage lysosomes. Anti‐CD41‐PLT‐VP16 is highly stable in a neutral environment, and the drug release rate can reach 85% at 35 h in an acidic environment. H) WB analysis was utilized to detect platelet function‐related proteins. I) WB detection of characteristic proteins on the surface of platelets from each group. J) The turbidimetric assay was utilized to determine platelet aggregation. The control group was untreated platelet‐rich plasma and PPP was platelet‐poor plasma, which had an absorbance value of 0 in the agglutination assay. The absorbance was measured after the addition of the procoagulant ADP to the remaining four groups, except for the control group, and the addition of the anti‐CD41‐PLT‐VP16 had no effect on platelet agglutination. K) Zeta potentials of the PLT, anti‐CD41‐PLT, and anti‐CD41‐PLT‐VP16 groups. ^***^ *p *< 0.001, ns = not significant.

Inspired by PLT in peripheral blood (PB) of ITP patients are more likely to be phagocytosed by macrophages, we simulated this pathological phenomenon and constructed mAb‐opsonized anti‐CD41‐PLT and loaded them with VP16. Morphological analysis by scanning electron microscopy (SEM) revealed no significant changes in the structure of the anti‐CD41‐PLT and anti‐CD41‐PLT‐VP16 compared to that of normal PLTs (**Figure** **1**A). Dynamic light scattering (DLS) measurements revealed particle sizes of 2000 and 2100 nm before and after VP16 loading, respectively (Figure 1B and *p* > 0.05). Confocal laser scanning microscopy (CLSM) revealed abundant expression of anti‐CD41 on the surface of PLTs extracted from ITP patients, which was absent in normal PLTs (Figure 1C). Flow cytometry (FCM) analysis further confirmed an antibody binding rate of 56% in sensitized PLTs (Figure 1D). VP16 was effectively loaded into anti‐CD41‐PLTs by the cyanide linkage method, and high‐performance liquid chromatography (HPLC) was used to measure the drug concentration. The results showed that the drug loading rate and encapsulation efficiency of anti‐CD41‐PLT‐VP16 were 41% and 15%, respectively (Figure 1E,F). The effective drug loading provided a foundation for further in vitro cell experiments and in vivo treatment of HLH. In this study, buffer solutions at pH 7.4 and 5.5 were used to simulate the physiological circulation and acidic environments of macrophage lysosomes. The results showed that the release efficiency of anti‐CD41‐PLT‐VP16 in PBS at pH 7.4 was low, with only 35% of the drug released at 36 h. However, in pH 5.5 PBS, the release rate of VP16 reached 85% (Figure 1G and *p* < 0.001). The ability of anti‐CD41‐PLT‐VP16 to effectively release drugs in acidic environments is beneficial for suppressing macrophage activity. Next, the biological characteristics of normal PLTs, anti‐CD41‐PLTs, and anti‐CD41‐PLT‐VP16 were compared. The Western blot (WB) results indicated no difference in the expression of the characteristic proteins CD41 and CD61 among the three groups. High IgG levels were associated with anti‐CD41 in the anti‐CD41‐PLT and anti‐CD41‐PLT‐VP16 groups (Figure 1H,I). The PLT aggregation test and zeta potential also demonstrated that the PLTs in the anti‐CD41‐PLT‐VP16 group possessed a similar aggregation function and charge to the normal PLTs (Figure 1J,K and *p* > 0.05). All the above results verified the successful extraction of anti‐CD41‐PLTs and the effective loading of VP16. Moreover, maintaining the biological structure and function of anti‐CD41‐PLT‐VP16 provides advantages for its effect on systemic circulation.

### 2.2 Anti‐CD41‐PLT‐VP16 Can Target Macrophages and Exert Superior Antimacrophage Effects In Vitro

**Figure 2:**
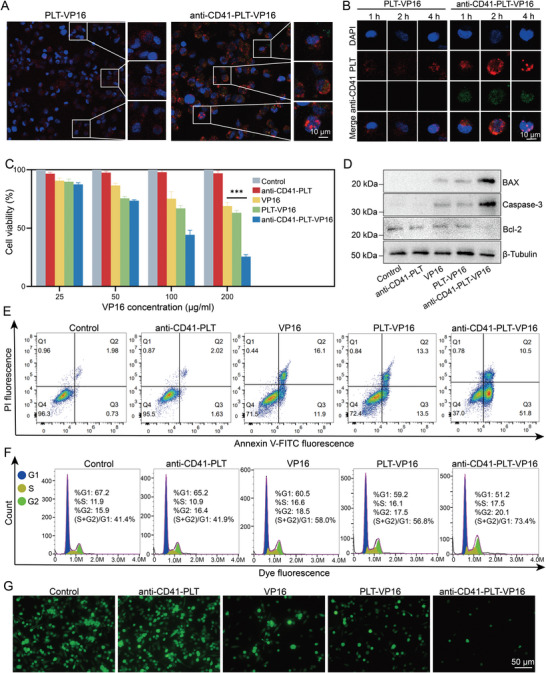
The effect of anti‐CD41‐PLT‐VP16 on macrophages. A) CLSM observation of phagocytosis of PLT‐VP16 and anti‐CD41‐PLT‐VP16 by macrophages. (PLT: red, DAPI: blue, bar = 10 µm) B) Phagocytosis of PLT‐VP16 and anti‐CD41‐PLT‐VP16 by macrophages was photographed by CLSM after 1, 2, and 4 h of administration. (DAPI: blue, PLT: red, anti‐CD41: green, bar = 10 µm) C) CCK‐8 measurement of macrophage viability in different treatment groups after 12 h of administration. D) WB analysis was used to detect changes in the levels of mitochondrial apoptosis pathway‐related proteins, including Bax, Caspase‐3, and Bcl‐2, in the different treatment groups after 12 h of administration. E) FCM with Annexin V‐FITC/PI was used to detect the apoptosis of macrophages in different treatment groups 12 h after drug administration. F) Cycle detection was performed on macrophages from different treatment groups after 12 h of drug administration, and an increase in (S+G2)/G1 could indicate cycle arrest due to blockage of DNA replication. G) ROS levels were detected in macrophages from each treatment group 12 h after administration and observed by fluorescence microscopy. The green fluorescence intensity indicates the level of ROS in the cells. Bar = 50 µm. ^**^ *p* < 0.01.

We further investigated the ability of macrophages to engulf anti‐CD41‐PLT‐VP16 actively. After coincubating macrophages with PLT‐VP16 or anti‐CD41‐PLT‐VP16 for 4 h, the red fluorescence signal representing anti‐CD41‐PLT‐VP16 was significantly stronger in the macrophages than in the other groups. Furthermore, as time progressed, the red fluorescence signal gradually increased. In contrast, the macrophages coincubated with the normal PLTs did not show a progressive increase in the internal fluorescent signal (**Figure** **2**A,B). Therefore, the presence of anti‐CD41 is crucial for targeting macrophages with anti‐CD41‐PLT‐VP16. To assess the synergistic effect of anti‐CD41‐PLTs and VP16 on macrophages, this study performed CCK‐8 assays to evaluate the impact of different treatments on macrophages (Figure 2C). After 12 h of treatment, the cell viability in the anti‐CD41‐PLT‐VP16 group was significantly reduced compared to that in the VP16 group and other PLT groups, which had a value of only 16%. The IC50s of the VP16 treatment group and the anti‐CD41‐PLT‐VP16 group were 6 and 3 µg mL^−1^, respectively (Figure S1A, Supporting Information). Moreover, the decrease in cell viability may be caused by the activation of the mitochondrial apoptosis‐related pathway (Figure 2D; Figure S1B, Supporting Information, *p* < 0.01). Subsequently, the therapeutic effects of different treatments were analyzed by FCM, as shown in the apoptosis graph (Figure 2E; Figure S1C, Supporting Information). Compared to the 6% apoptosis rate in the control group, the apoptosis rate in the anti‐CD41‐PLT‐VP16 treatment group reached a maximum of 71%, ≈1.26 times greater than that in the free VP16 group.

We conducted relevant experiments to evaluate whether the killing effect of the anti‐CD41‐VP16 agent is related to the production of reactive oxygen species (ROS) in macrophages or cell cycle arrest. The FCM results of the cell cycle (Figure 2F) revealed that free VP16 blocked cells in the G0/G1 phase (53.33%). This result is mainly due to VP16, a DNA topoisomerase inhibitor, which can hinder DNA replication and lead to cell cycle arrest.^[^ ^23^, ^24^, ^25^ ^]^ In the anti‐CD41‐PLT‐VP16 group, the treatment had even greater effects, as this system blocked more macrophages in the G0/G1 phase (67.31%). The ROS levels of cells in each treatment group were detected. The ROS levels in macrophages after treatment with anti‐CD41‐PLT‐VP16 were significantly reduced and were ≈1.95 times and 2.28 times lower than those in the VP16 group and PLT‐VP16 group, respectively (Figure 2G; Figure S1D, Supporting Information, *p* < 0.05). Due to the direct correlation between ROS levels and macrophage activity,^[^ ^26^, ^27^, ^28^ ^]^ the results of CLSM fluorescence imaging suggest that anti‐CD41‐PLT‐VP16 can most effectively inhibit macrophages. Activated macrophages are generally classified into M1‐like macrophages and M2‐like macrophages. M1 macrophages are primarily involved in proinflammatory responses, while M2 macrophages are mainly involved in anti‐inflammatory responses. Previous studies have shown that anti‐CD4‐PLT‐VP16 can effectively inhibit macrophage activation. Therefore, this study further analyzed the activation status of macrophages treated with anti‐CD4‐PLT‐VP16 using FCM. The results indicated that the proportion of M2 macrophages was 1.18 times greater in the anti‐CD41‐PLT‐VP16‐treated group than in the control group. In contrast, the proportion of M1 macrophages was 0.87 times that of the control group (Figure S1E, Supporting Information). This finding suggested that anti‐CD4‐PLT‐VP16 can treat this disease by inhibiting macrophage activity and promoting the generation of anti‐inflammatory M2 macrophages.

Overall, anti‐CD41‐PLT‐VP16 can effectively inhibit macrophage activity and accelerate apoptosis by arresting the cell cycle of macrophages and inhibiting intracellular ROS production. Notably, targeted reduction of macrophage activity is critical for treating HLH. Therefore, anti‐CD41‐PLT‐VP16 has an excellent therapeutic effect on HLH.

### 2.3 Anti‐CD41‐PLT‐VP16 Can Efficiently Alleviate HLH and Inhibit Cytokine Storms in a Humanized HLH Mouse Model

To further simulate the human physiological environment and investigate the effect and possible mechanism of anti‐CD41‐PLT‐VP16, this study constructed a humanized HLH mouse model. The internal structures of the liver and spleen tissues were loosely organized in the model group (Figure S2A, Supporting Information). F4/80 immunohistochemical images showed many macrophages infiltrating the liver and spleen. Erythrophagocytosis was detected in the bone marrow (BM) and PB but not in the controls. Compared to the unmodeled mice, the liver and spleen volumes of the mice with HLH were significantly greater (Figure S2B–E, Supporting Information). In addition, the results of PB imaging, cytokine assays, and biochemical assays showed that the mice in the modeling group exhibited significantly suppressed BM hematopoiesis (Figure S2F,G, Supporting Information, *p* < 0.001) and high IL‐1β, IL‐6, and TNF‐α levels, indicating the production of cytokine storms (Figure S2H, Supporting Information, *p* < 0.01). Moreover, the model mice exhibited elevated triglyceride (TG) levels (Figure S2I, Supporting Information, *p* < 0.05). These findings support the successful construction of the HLH mouse model in the present study.

**Figure 3:**
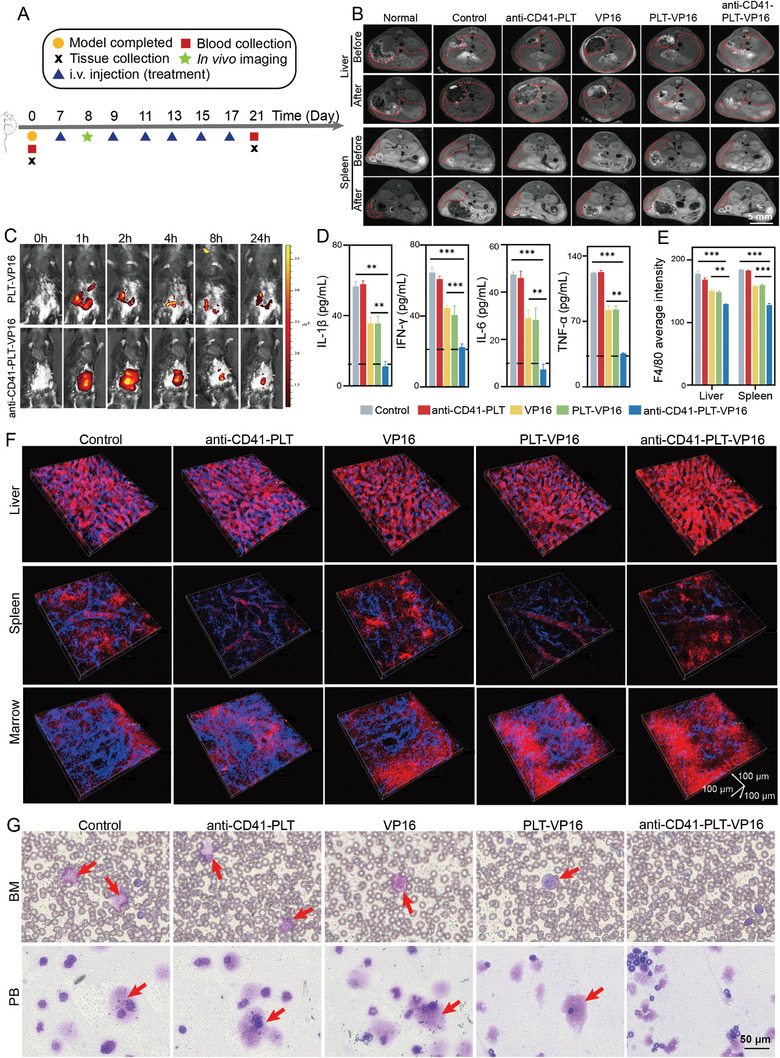
The therapeutic effect of anti‐CD41‐PLT‐VP16 in humanized mice with HLH. A) Treatment schedules for the different groups of the humanized HLH model mice. B) MR images of liver and spleen tissues from mice in different treatment groups before and after treatment (*n* = 6). The liver and spleen were photographed separately at the same level, and the regions circled by the red dotted line are the liver and spleen tissue. Bar = 5 mm. C) In vivo imaging of small animals showing the circulation and enrichment of anti‐CD41‐PLT‐VP16 in the tail vein over time after the injection of Cy5‐labeled anti‐CD41‐PLT‐VP16 (*n* = 6). D) Measurement of IL‐1β, IFN‐γ, IL‐6, and TNF‐α levels in the PB after treatment of mice in different treatment groups (*n* = 6). Dashed lines indicate normal values. E) Quantitative results of F4/80 in liver and spleen tissues determined by IHC after treatment of mice in different treatment groups (*n* = 6). F) IVM real‐time in vivo imaging of liver, spleen, and BM macrophage activity in different treatment groups of mice 12 h after the corresponding treatments. The blue fluorescence intensity indicates macrophage activity. (Blood: red, cathepsin K probe: blue, *n* = 6). G) Wright‐Giemsa staining of PB and BM from mice in different treatment groups. Arrows show hemophagy (*n* = 6). Bar = 50 µm. ^*^ *p* < 0.05, ^**^ *p* < 0.01, ^***^ *p* < 0.001.

Based on the humanized mice with HLH described above, we administered different treatments and ensured that the VP16 concentration was the same (**Figure** **3**A). This study applied magnetic resonance imaging (MRI) to preliminarily compare the changes in the liver and spleen sizes of each group of mice before and after treatment. After treatment, the anti‐CD41‐PLT‐VP16 group showed a significant reduction in the size of the liver and spleen (Figure 3B; Figure S3A,B, Supporting Information). Next, this study explored the biological distribution of anti‐CD41‐PLT‐VP16 in mice. The results showed that the concentration of anti‐CD41‐PLT‐VP16 peaked in liver and spleen tissues at 1–2 h, and CD41‐PLT‐VP16 was present in the circulation until 24 h (Figure 3C; Figure S3C, Supporting Information).

]The cytokine storm plays a critical role in HLH and is characterized by the expression of IFN‐γ, IL‐6, TNF‐α, and IL‐1β.^[^ ^9^, ^10^ ^]^ Therefore, we first evaluated the inhibitory effect of anti‐CD41‐PLT‐VP16 on cytokine storms. Compared to those in the other treatment groups, the expression levels of four cytokines, namely, IL‐1β, IFN‐γ, IL‐6, and TNF‐α, were significantly lower in the anti‐CD41‐PLT‐VP16 group of humanized mice with HLH (Figure 3D, *p* < 0.05). Moreover, the coagulation function of the mice in the anti‐CD41‐PLT‐VP16 group was substantially improved, and the serum ferritin level was significantly decreased. Furthermore, the effect of anti‐CD41‐PLT‐VP16 on TG levels was less pronounced than the effect caused by VP16 alone, which substantially increased the TG level (Figure S3D,E, Supporting Information, *p* < 0.05). In addition, compared with those in the group treated with VP16 alone, the complete blood counts in the anti‐CD41‐PLT‐VP16 group were significantly greater, and the elevations in Hb, red blood cell (RBC), white blood cell (WBC), and PLT counts were significant (Figure S3F, Supporting Information, *p* < 0.05). After treatment, reduced hepatic and splenic cell damage was observed in the anti‐CD41‐PLT‐VP16 group (Figure S3G, Supporting Information). In addition, after treatment with CD41‐PLT‐VP16, the percentage of F4/80+ inflammatory cells according to immunohistochemistry (IHC) of liver and spleen tissue slices from mice with HLH was reduced by 23% and 18%, respectively, compared with that in the VP16 and PLT‐VP16 treatment groups (Figure 3E; Figure S3H, Supporting Information).

Macrophages can secrete cathepsin K, and the amount of cathepsin K secretion increases with increased macrophage activity. This finding implies that the level of surface cathepsin K on macrophages can reflect the strength of macrophage activity. Therefore, this study utilized a cathepsin K probe to label macrophages in a humanized HLH mouse model. Using an intravital microscopy (IVM) imaging system, this study dynamically and in real‐time observed changes in macrophage activity within mice. Results showed that the anti‐CD41‐PLT‐VP16 treatment effectively suppressed macrophage activity in the liver, spleen, and BM. The inhibitory strength was 2.8 times greater than that of the PLT‐VP16 group (Figure 3F; Figure S3I,J, Supporting Information). After performing BM imaging on the treated mice, Results showed the disappearance of hemophagocytes in the anti‐CD41‐PLT‐VP16 group, while the other groups still exhibited varying degrees of hemophagocytes (Figure 3G). As a highly effective immunotherapeutic agent, CD41‐PLT‐VP16 can remove cytokines from the circulatory system and the liver, spleen, and BM. This treatment inhibited the activation of macrophages and helped restore normal liver and hematopoietic function.

**Figure 4:**
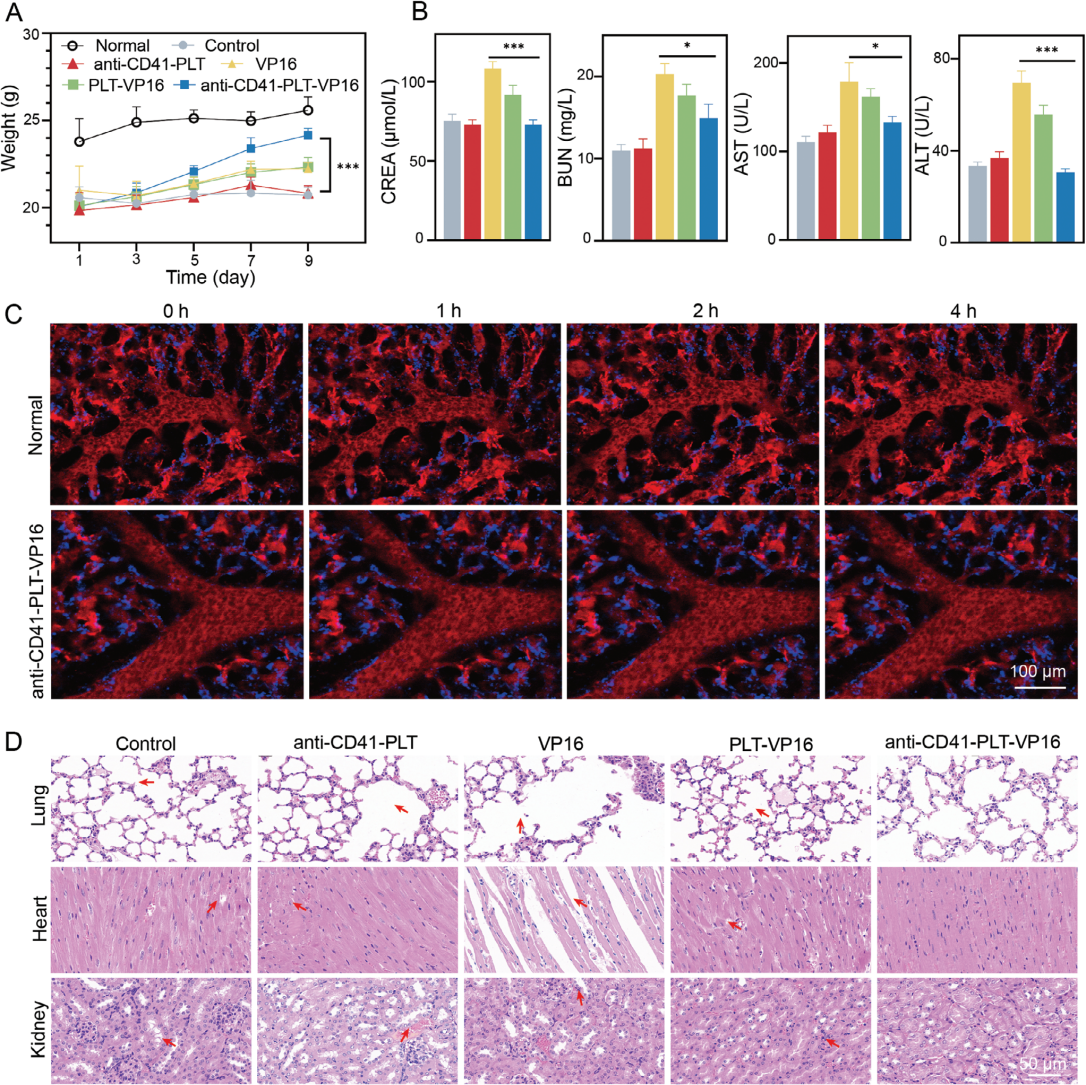
Detection of the biosafety of anti‐CD41‐PLT‐VP16. A) Changes in the body weight of mice in different treatment groups (*n* = 6). The weight of the anti‐CD41‐PLT‐VP16 group after treatment converged on that of normal mice before modeling. B) Renal function measurements, including creatinine (CREA) and nitrogen (BUN), in mice in different treatment groups after treatment (*n* = 6). Liver function measurements, including aspartate aminotransferase (AST) and alanine aminotransferase (ALT) levels, in mice in different treatment groups after treatment (*n* = 6). C) In vivo real‐time imaging of large blood vessels in mice at 0, 1, 2, and 4 h after tail vein injection of anti‐CD41‐PLT‐VP16 (blood: red, cathepsin K probe: blue, *n* = 3, bar = 100 µm). D) H&E staining of mouse lung, heart, and kidney tissues in different treatment groups (*n* = 6). Bar = 50 µm. ^*^ *p* < 0.05, ^***^ *p *< 0.001.

VP16 is a first‐line drug for treating HLH, but it often causes damage to multiple tissues and organs because of its lack of specificity. Previous results confirmed that anti‐CD41‐PLT‐VP16 has increased macrophage targeting properties, so this study explored the biosafety of anti‐CD41‐PLT‐VP16 in vivo. Mice in the anti‐CD41‐PLT‐VP16 group had a much greater body weight increase during treatment than those in the VP16 group (**Figure** **4**A, *p* < 0.001). Additionally, the nephrotoxicity and hepatotoxicity of anti‐CD41‐PLT‐VP16 were significantly lower than those of VP16 (Figure 4B, *p* < 0.05). PLTs can easily aggregate and form blood clots. We must consider whether administering anti‐CD41‐PLT‐VP16 could lead to vascular obstruction in mice. Therefore, the major blood vessels of the mice were dynamically observed in real time using IVM at 0, 1, 2, and 4 h after intravenous injection of anti‐CD41‐PLT‐VP16. The blood flow in the mice was normal, and there was no formation of blood clots (Figure 4C). Furthermore, no tissue damage was observed by H&E staining of the experimental mice's lungs, heart, or kidneys (Figure 4D). The above experimental results showed that, compared with VP16 monotherapy for HLH, anti‐CD41‐PLT‐VP16 has better biosecurity and can significantly reduce the toxic side effects in the organism. The excellent safety of these agents provides support for further clinical trials.

### 2.4 Anti‐CD41‐PLT‐VP16 May Inhibit Cytokine Storms by Reducing Macrophage Activation and Indirectly Decreasing Other Immune Cells' Activity

**Figure 5:**
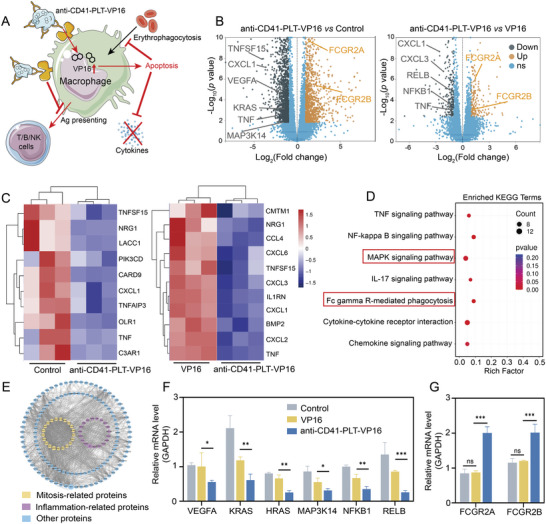
Mechanism of inhibition of inflammation‐associated cytokine storm by anti‐CD41‐PLT‐VP16. A) Schematic illustration of the in vivo process in which anti‐CD41‐PLT‐VP16 prevents antigen presentation by occupying the Fc receptor on the surface of macrophages in HLH through ADCP; after being phagocytosed by macrophages, anti‐CD41‐PLT‐VP16 can induce the apoptosis of macrophages and inhibit cytokine release. B) Volcano plots show the upregulation and decrease in gene expression. The expression of some of the genes associated with Ras/MAPK, inflammation, and FcγR‐mediated phagocytosis are labeled in the figures (*n* = 3). C) Heatmap showing the expression of cytokine‐related genes in each group (*n* = 3, *p* < 0.05). D) KEGG enrichment analysis comparing the anti‐CD41‐PLT‐VP16 group with the VP16 group. Bubble plots showing the pathways in which differentially expressed proteins were concentrated (*p* < 0.05). E) Differentially expressed genes in the anti‐CD41‐PLT‐VP16 group versus the control group were analyzed by PPI (*p* < 0.05). F) Quantitative real‐time PCR (qPCR) was performed on the control, VP16, and anti‐CD41‐PLT‐VP16 groups, and the graph shows the expression levels of genes involved in the Ras‐MAPK pathway in each group. G) qPCR results showing the expression levels of genes involved in the FcγR‐mediated phagocytosis pathway. ^*^ *p* < 0.05, ^**^ *p* < 0.01, ^***^ *p* < 0.001.

Next, we explored the possible mechanisms by which anti‐CD41‐PLT‐VP16 treats HLH and inhibits cytokine storms (**Figure** **5**A). After drug administration, this study performed transcriptome sequencing on macrophages from the control, VP16, and anti‐CD41‐PLT‐VP16 groups. The expression profiles of the three groups were significantly different. The volcano plot shows the changes in gene levels in the remaining two groups compared to those in the anti‐CD41‐PLT‐VP16 group; results showed a significant decrease in the expression of genes associated with the Ras/MAPK pathway and inflammation, while the expression of genes associated with FcγR‐mediated phagocytosis increased (Figure 5B). The heatmap shows the differential expression of inflammatory cytokine‐related pathway components, and the anti‐CD41‐PLT‐VP16 group had low expression levels of relevant genes, which indicates that anti‐CD41‐PLT‐VP16 was more effective at reducing cytokine storms in individuals with HLH (Figure 5C). In addition, KEGG and protein‒protein interaction (PPI) enrichment analyses showed that anti‐CD41‐PLT‐VP16 might inhibit the Ras/MAPK and NF‐κB signaling pathways and promote the ADCP process (Figure 5D,E). Activated Ras/MAPK and NF‐κB pathways are related to the triggering of proinflammatory cytokine production.^[^ ^29^, ^30^, ^31^ ^]^ Anti‐CD41‐PLT‐VP16 may competitively inhibit antigen presentation by macrophages through the activation of ADCP, thus preventing the abnormal activation and cytokine release of T cells, B cells, and NK cells. To verify the above results, we determined the expression levels of genes involved in the Ras/MAPK and FcγR‐mediated phagocytosis pathways, and the results confirmed that the expression of genes involved in the Ras/MAPK pathway was reduced (Figure 5F). Furthermore, the gene expression levels of FCGR2A and FCGR2B, key molecules mediating FcγR‐mediated phagocytosis, were elevated 1.8‐ to 2.1‐fold in the anti‐CD41‐PLT‐VP16 group (Figure 5G).

Anti‐CD41‐PLT‐VP16 may inhibit inflammation‐associated cytokine storms by suppressing the expression of a series of genes in the pathway above, ultimately inhibiting inflammation‐associated cytokine storms (Figure 5F,G). In summary, anti‐CD41‐PLT‐VP16 may inhibit the Ras/MAPK pathway and compete to inhibit the remaining FcγR‐related activity through FcγR‐mediated phagocytosis, which may inhibit cytokine storms not only by macrophages but also by other immune cells.

### 2.5 Anti‐CD41‐PLT‐VP16 Alleviates HLH in Patients

Given that less than half of the patients who previously received VP16 in combination with other supportive treatments achieved remission, new treatment approaches to cure HLH are needed. Based on comprehensive validation of the efficacy and safety of anti‐CD41‐PLT‐VP16, this study performed the first clinical trial of platelet‐based therapy for HLH in Asia. We designed and initiated a single‐arm clinical trial (phase Ib) to evaluate the simultaneous administration of dexamethasone and cyclosporine with anti‐CD41‐PLT‐VP16 in HLH patients (reference number: 3923‐435‐02).

**Figure 6:**
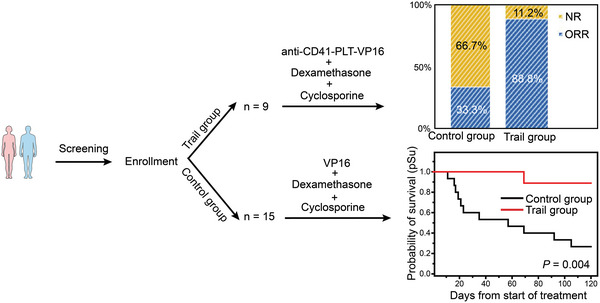
Schematic diagram of the preliminary clinical trial. The control group was normally treated patients, and VP16 at the time of treatment was replaced with anti‐CD41‐PLT‐VP16 in equal final concentration in the trial group. A significantly higher disease remission rate was found in the anti‐CD41‐PLT‐VP16 group. “ORR” = Objective response rate; “NR” = No response.

In the initial phase, this study recruited 9 HLH patients who showed hematological evidence of disease progression while receiving dexamethasone and cyclosporine in combination with anti‐CD41‐PLT‐VP16 treatment (Table S1, Supporting Information). After treatment, 88.8% of the patients achieved an overall response rate (ORR), and 11.2% had no remission (NR). The 120‐day survival rate of all included patients was observed, and the results showed that the percentage of patients in the experimental group was as high as 88.89%, and only one patient died on the 68th day after treatment. In comparison, the 120‐day survival rate of the control group was only 26.67% (**Figure** **6** and Table S2). The nonremission rate in the patients treated with a combination of dexamethasone, cyclosporine, and VP16 was as high as 46.7%. Given the small sample size of this phase Ib trial, we must avoid generalizing conclusions about the efficacy of this combination treatment and plan to conduct a larger phase II trial to assess the benefits of using anti‐CD41‐PLT‐VP16 in patients receiving combination therapy. These studies will contribute to the clinical‐scale promotion of anti‐CD41‐PLT‐VP16.

## 3 Discussion

HLH is a hyperinflammatory response syndrome caused by abnormalities in the body's immune system, with clinical manifestations such as phagocytosis of blood cells, hemorrhage, impairment of liver and spleen functions, and an extremely high morbidity and mortality rate.^[^ ^32^, ^33^, ^34^ ^]^ Several studies have shown that abnormal activation of the monocyte‐macrophage system plays an essential role in the development of this disease, which causes hemophagocytosis and the production and release of many cytokines, resulting in a cytokine storm.^[^ ^35^, ^36^ ^]^ Therefore, macrophages are critical target cells for the treatment of HLH. A therapeutic method to target abnormally activated macrophages in HLH and to control cytokine storms is urgently needed.^[^ ^37^, ^38^, ^39^ ^]^ VP16, a basic therapeutic regimen, is currently the first‐line drug for treating HLH.^[^ ^40^, ^41^, ^42^, ^43^ ^]^ VP16 targets DNA topoisomerase‐2, acts selectively on the DNA of rapidly dividing cells, and has a rapid onset of action on abnormally activated macrophages.^[^ ^23^, ^24^, ^25^, ^44^, ^45^, ^46^ ^]^ However, VP16 lacks specificity, and its clinical efficacy is not optimal. This issue is because VP16 is not easily soluble in water, and achieving an effective therapeutic dose is difficult.^[^ ^47^, ^48^, ^49^, ^50^ ^]^ Therefore, precision immunotargeted therapy with high specificity and low toxicity is urgently needed to treat HLH.^[^ ^37^, ^38^, ^39^, ^51^ ^]^

ADCP plays a critical regulatory role in macrophage endocytosis.^[^ ^52^, ^53^, ^54^ ^]^ Our Previous study showed that the anti‐CD41‐PLTs present in ITP patients were phagocytosed by macrophages much more efficiently than normal PLTs. In this study, we mimicked the pathological mechanisms of ITP patients to design and construct the anti‐CD41‐PLT‐VP16 living therapeutic platform for the treatment of HLH.

Results showed that anti‐CD41‐PLT‐VP16 can effectively target and is phagocytosed by macrophages in vitro. Moreover, this treatment effectively reduced macrophage activity. In addition, anti‐CD41‐PLT‐VP16 effectively increased the inhibitory effect of VP16 on topoisomerase II and inhibited cell cycle progression in the late S and early G2 phases. Furthermore, anti‐CD41‐PLT‐VP16 accumulated in the liver and spleen and crossed the bone‐blood barrier, affecting marrow macrophages. To further investigate the underlying mechanism, this study utilized transcriptome sequencing and found that anti‐CD41‐PLT‐VP16 significantly inhibited the expression of inflammation‐related cytokines. Moreover, this treatment may regulate inflammation‐associated cytokine expression by modulating the Ras/MAPK pathway, as supported by the qPCR results. The above mechanism for alleviating cytokine storms, such as reduced NF‐κB levels, has been similarly reported in other studies.^[^ ^55^, ^56^, ^57^ ^]^ In addition, as the ADCP effect of macrophages phagocytizing anti‐CD41‐PLT‐VP16 involves the FcγR, anti‐CD41‐PLT‐VP16 can competitively inhibit antigen presentation from macrophages to T and B cells, which may inhibit the activation of other immune cells and the ADCC effect. This finding may also explain why anti‐CD41‐PLT‐VP16 inhibits the hyperinflammatory syndrome caused by the abnormal activation of immune cells.

Based on in vitro experiments, we constructed a humanized HLH mouse model and verified the therapeutic effect of anti‐CD41‐PLT‐VP16 on this model. This study explored the enrichment of anti‐CD41‐PLT‐VP16 in liver and spleen tissues and confirmed that anti‐CD41‐PLT‐VP16 significantly reduced macrophage activity and inhibited cytokine storms. Increased targeting of anti‐CD41‐PLT‐VP16 could effectively reduce the toxic side effects of VP16 on nontarget organs. Finally, to translate scientific knowledge from bench to bedside, this study explored the possibility of practically applying anti‐CD41‐PLT‐VP16 in the clinic. We are enrolling eligible patients in clinical trials, and the clinical data collected thus far indicate that VP16 has limited therapeutic effects on HLH and exhibits toxic side effects. We are conducting the first clinical study in China to apply the platelet‐based living therapeutics platform in practice, and a clinical trial of anti‐CD41‐PLT‐VP16 for the treatment of HLH is in progress.

In summary, these findings provide new clinical ideas for platelet‐based living therapeutics platforms targeting macrophages and new immunotherapeutic strategies for controlling cytokine storms and open a new chapter in clinical research on platelet‐based living therapeutics platforms.

## 4 Experimental Section

### ITP Patient Screening

Eligible patients with ITP were adult age (≥18 years old) and had primary ITP. IgG antibodies were detected in the peripheral blood of patients by indirect monoclonal antibody‐specific immobilization of platelet antigens (MAIPA), including GPIIb/IIIa, GPIb/IX, and GPIa/IIa, and finally screened for patients with GPIIb/IIIa, i.e., CD41 autoantibodies. The patient's peripheral blood was tested by direct monoclonal antibody‐specific immobilization of platelet antigens (MAIPA) for IgG antibodies, including GPIIb/IIIa, GPIb/IX, and GPIa/IIa, and was screened for the presence of GPIIb/IIIa, i.e., CD41 autoantibodies.

### Preparation of Anti‐CD41‐PLT‐VP16

The PLT concentrate was centrifuged at 1500 × *g* for 10 min at 23 ± 1 °C to pellet the PLTs. PLTs were then gently resuspended in VP16 solution at different concentrations (Sunnyhope, China) and solubilized in PBS. The preparation was incubated gently on a suspension mixer (Yihder, China) at 30 ± 5 rpm for 1 h at 37 ± 1 °C. Excess VP16 was removed by centrifugation at 1500 × g for 10 min at 23 ± 1 °C. The pellets were gently resuspended in PBS to generate fresh VP16‐loaded PLTs or in 6% DMSO (Beyotime Biotechnology, China) to prepare cryopreserved VP16‐loaded PLTs. Anti‐CD41 (Beyotime Biotechnology, China) was added to the surface of PLTs by adding 5 µL of anti‐CD41 to 100 µL of PLT suspension and incubating for 20 min at 4 °C.^[^ ^19^, ^20^ ^]^ Excess anti‐CD41 was removed by centrifugation at 1500 × g for 10 min at 23 ± 1 °C. The resulting solution was washed three times with PBS.

### Characterization of Anti‐CD41‐PLT‐VP16

PLTs, PLT‐VP16, and anti‐CD41‐PLT‐VP16 were photographed by SEM. DLS was used to determine the average particle size of PLTs. Colocalization of the anti‐CD41‐PLT‐VP16 components was assessed under a CLSM, and the PLTs were stained with phalloidin (MedChemExpress, US) and imaged by CLSM with FITC‐conjugated anti‐CD41 (Thermo Fisher Scientific, US). HPLC was used to determine the encapsulation rate of anti‐CD41‐PLT‐VP16 and its release efficiency under different pH conditions. The calculation formulas are as follows: encapsulation rate (EE) = (content of encapsulated drug in the carrier/total amount of drug) × 100% and drug loading rate = (drug mass/total mass of the carrier) × 100%.^[^ ^58^, ^59^ ^]^ A turbidimetric assay was used to determine the agglutination function of PLTs, and the control group consisted of PLT‐rich plasma not stimulated by a procoagulant. WB analysis was used to measure the PLT surface marker protein content. The percentage of anti‐CD41 attachment on PLTs was calculated using FCM (antibodies purchased from Beyotime Biotechnology, China).

### Cell Lines and Culture

The Research Department of Nanjing Drum Tower Hospital provided the human monocyte cell line THP‐1. The cells were cultured in Roswell Park Memorial Institute (RPMI) 1640 medium (Gibco, US) supplemented with 10% fetal bovine serum (Gibco, US), penicillin, and streptomycin at a final concentration of 10 U mL^−1^. The cell concentration was controlled at (1–10) × 10^5^ mL^−1^, the cells were cultured in a 37 °C, 5% CO_2_ incubator, and the culture medium was changed every 2–3 days. THP‐1 cells were induced with a medium supplemented with PMA (MedChemExpress, US), and macrophages were obtained after 48 h. The macrophages were cultured under the same conditions as those used for THP‐1 cells.

### Cellular Experiments

The localization of macrophages and platelets and the results of anti‐CD41 staining were visualized with DAPI (Beyotime Biotechnology, China), phalloidin, and FITC. A confocal microscope was used to determine the phagocytic effect of the macrophages on the living therapeutic platform. Cell viability was determined with a CCK‐8 kit (Beyotime Biotechnology, China). Cell ROS levels were determined by FCM and fluorescence microscopy using a ROS kit (Abbkine, China). The macrophages from the different treatment groups were stained with an Annexin V‐FITC/PI Apoptosis Kit (Abbkine, China). FCM determined the degree of apoptosis, and the cells were defined as early and late apoptotic cells.

### Targeted Recognition and Cell Internalization

THP‐1 cells were inoculated in 15 mm confocal dishes at a density of 1 × 10^6^ cells per well and induced for 48 h. PLT‐VP16 and anti‐CD41‐PLT‐VP16 (VP16 concentration: 50 µg mL^−1^) were added to the confocal culture dishes and incubated with the cells simultaneously. The treated cells were washed three times with PBS and then stained with DAPI (Ex/Em = 359/461 nm, Beyotime Biotechnology, China) for 10 min. Then, the treated cells were fixed with 4% paraformaldehyde and washed three times with PBS. Finally, the cells were observed via CLSM.

### Cell Viability and Apoptosis Test

In the in vitro cytotoxicity assay, THP‐1 cells were inoculated into 96‐well plates at a density of 1 × 10^4^ cells per well. After 48 h in the induction medium, the medium was discarded. Subsequently, the cells were treated with PLTs, VP16, PLT‐VP16, and anti‐CD41‐PLT‐VP16 (for which the concentration gradient of VP16 was 25, 50, 100, or 200 µg mL^−1^, respectively) and incubated for a total of 12 h. Tissue culture plates without samples were used as controls. Cell viability was quantified using a standard CCK‐8 assay (Beyotime Biotechnology, China), and the data represent the average of three parallel measurements. For analysis of apoptosis in the different treatment groups (the treatment groups and added concentrations were consistent with the CCK‐8 assay), the cells were digested with EDTA‐free trypsin and centrifuged at 2000 rpm for 5 min to collect and washed; afterward, 500 µL of binding buffer was added to gently resuspend the cells by swirling; 5 µL of Annexin V‐FITC (Abbkine, China) was added to the cells, which were mixed by swirling; 5 µL of PI was added to the cells, which were mixed by swirling gently; and then, 5 µL of PI was added to the cells, which were gently mixed by swirling. Then, 5 µL of Annexin V‐FITC was added and mixed, followed by 5 µL of PI (Abbkine, China) and mixing. The mixture was incubated at room temperature in the dark for 10 min and detected by FCM.

### Cell Cycle Test

Cells (5 × 10^5^) from different treatment groups were collected in centrifuge tubes, washed with 1 mL of precooled PBS supplemented with ≈1 mL of precooled anhydrous ethanol, gently swirled to mix well, and fixed at 4 °C for 4 h. One milliliter of precooled PBS was added, and the supernatant was discarded. Then, 100 µL of RNase A was added, the cells were well suspended, and the cells were incubated in a water bath at 37 °C for 30 min. Then, 400 µL of PI solution (Beyotime Biotechnology, China) was added and mixed well. The samples were incubated at 4 °C for 30 min in the dark and tested on a machine, and the DNA content was analyzed using analysis software.

### Cellular ROS

For verification of the level of ROS in the cells after different treatments, 1 × 10^6^ THP‐1 cells were cultured in 15 mm confocal dishes for 48 h of induction culture and were then treated with PBS, PLTs, VP16, PLT‐VP16, or anti‐CD41‐PLT‐VP16 (all with a VP16 concentration of 50 µg mL^−1^). After coincubation for 12 h at 37 °C, the single linear oxygen probe DCFH‐DA (10 µm, λex = 488 nm, λem = 525 nm, Abbkine, China) was added, and the cells were incubated for 20 min. The cell culture medium was discarded, and the cells were rinsed twice with PBS. CLSM was used to determine the fluorescence of DCF.

### Macrophage Typing

Macrophages were stained with FITC‐conjugated anti‐CD68, APC‐conjugated anti‐CD163, and PE‐conjugated anti‐CD86 (BioLegend, US), and FCM was used to differentiate the M1 and M2 polarization of macrophages, in which double‐positive CD68+ and CD86+ macrophages were defined as M1 macrophages and CD68+ and CD163+ double‐positive macrophages were defined as M2 macrophages.

### The Preparation of Transcriptome Sequencing

After the cells were treated with PBS, VP16, and anti‐CD41‐PLT‐VP16 for 12 h (final concentration of VP16 200 µg mL^−1^), the culture medium was removed and washed once quickly with PBS. 1 mL of TRIzol was added to each well and then blown repeatedly. The above solution was transferred to RNase‐free 1.5 mL centrifuge tubes and blown repeatedly until no clumped cell mass was visible. Liquid nitrogen snap‐frozen and ready for transcriptome sequencing.

### Humanized HLH Mouse Model

Female NSG mice (20–24 weeks old) were purchased from Nanjing Qinglongshan Animal Breeding Farm (Nanjing, China). The ethical review number of this experiment was 2020≈gl18. CD34+ hematopoietic stem cells were injected into NSG mice (*n* = 42) as described previously.^[^ ^60^, ^61^, ^62^ ^]^ The humanized model mice were randomly divided into six groups (*n* = 6), 5 of which were subjected to HLH modeling treatment, and the remaining group was injected with an equal dose of physiological saline. The modeling treatment lasted a total of 10 days, and each mouse was subcutaneously injected with 0.8 µg/100 µL IFN‐γ once a day and 500 µg/100 µL CpG ODN every other day.^[^ ^63^ ^]^ After modeling, mouse tissues and PB were obtained for experimental testing to verify the modeling results.

### In Vivo Experiments in Mice with HLH—In Vivo Fluorescence Imaging

To study the accumulation behavior of the carriers in vivo, we loaded a near‐infrared (NIR) dye, Cy5, into PLT‐VP16 with anti‐CD41‐PLT‐VP16. After tail vein injection of 100 µL of Cy5‐PLT‐VP16 with Cy5‐anti‐CD41‐PLT‐VP16, the mice were anesthetized by inhalation of isoflurane, and recordings were made using an IVIS system (λex = 645 nm, λem = 675 nm) at the indicated times (0, 1, 2, 4, 8, 24 h) for Cy5‐PLT‐VP16 or Cy5‐CD41‐PLT‐VP16 in vivo fluorescence images.

### In Vivo Experiments in Mice with HLH—Determination of Macrophage Viability In Vivo

After the last treatment, mice in different treatment groups were given the Pan‐Histoplasmin 680 Fluorescent Probe (concentration, 100 µL) by tail vein injection, and the macrophage activity inside the liver and spleen of in vivo mice was detected by the IVM system after 12 h. The activity of macrophages inside the liver and spleen was measured by the IVM system (IVISense Pan‐Cathepsin 680 Fluorescent Probe, PerkinElmer).^[^ ^62^, ^63^, ^64^, ^65^, ^66^ ^]^

### In Vivo Experiments in Mice with HLH—In Vivo Treatment Efficacy

After the successful generation of the mouse HLH model was verified, the remaining mice with HLH were treated. Mice with HLH were randomly divided into five groups (*n* = 6) and injected with equal doses of saline, PLTs, VP16, PLT‐VP16, anti‐CD41‐PLT‐VP16, or anti‐CD41‐PLT‐VP16 via the tail vein on days 1, 3, 5, 7, and 9, in which VP16‐containing fractions were controlled to be equal to their final concentrations (300 µg/100 µL). On the first day at the end of treatment, blood was collected from the mice for inflammatory cytokine analysis, routine blood tests, liver and kidney function tests, coagulation factor analysis, and TG analysis. The vital organs, including the liver and spleen, were removed, weighed, and imaged. BM and PB smears from mice in different treatment groups were collected and subjected to Wright's staining for phagocytosis analysis. The livers and spleens of mice in different treatment groups were fixed with 4% paraformaldehyde overnight, rinsed in PBS, dehydrated, paraffin‐embedded, and sectioned. IHC analysis was performed to observe F4/80 expression, which indicates the abnormal activation of macrophages. IHC was used to analyze changes in the liver and spleen tissues' TNF‐α and IL‐6 inflammatory factor levels.

### In Vivo Experiments in Mice with HLH—Safety Evaluation

Mouse body weights were measured throughout the treatment, and the weight change curves of the mice were plotted. On the first day after treatment, PLT‐VP16 and anti‐CD41‐PLT‐VP16‐treated mice were injected with 100 µL of FITC in the tail vein and anesthetized by inhalation of isoflurane, and the presence of thrombi in the portal vein of mice in both groups was observed by an IVM spectroscopy system (λex = 495 nm, λem = 520 nm). Heart, lung, and kidney tissues were collected for sectioning and H&E staining for each group of mice.

### Anti‐CD41‐PLT‐VP16 Treatment Study in HLH Patients

Informed consent was obtained from all study participants. Based on these preclinical data, we designed and opened a clinical trial in which patients with relapsed/refractory HLH (reference number: 3923‐435‐02). The trial was designed to test whether this newfound anti‐CD41‐PLT‐VP16 therapeutic platform could efficiently treat HLH. The efficacy was evaluated as objective response rate (ORR). Therapeutic administration in the clinical trial was based on HLH‐1994 therapeutic criteria for administration, in which the administration of the anti‐CD41‐PLT‐VP16 group changed the VP16 monotherapy into anti‐CD41‐PLT‐VP16 with the same final concentration of VP16, and the rest of the treatment was the same as HLH‐1994.

### Statistical Analysis

All experiments were repeated three times, and the statistical data are expressed as the mean ± standard deviation (mean ± SD). The Shapiro‒Wilk method was used to test whether the experimental data conformed to a normal distribution, and the Hartley method was used to test the homogeneity of variance. When the data conformed to a normal distribution, an independent sample *t*‐test was used, and ANOVA was used to compare three or more groups. When the data did not conform to a normal distribution, the Mann‒Whitney U test was used, and the K‐independent samples test was used for comparisons among three or more groups. When *p* < 0.05, the difference was considered significant. Statistical processing was performed with SPSS 22.0 software, and graphs were generated using GraphPad Prism 8 software.

### Ethics Statements

All the experimental animals were purchased from Nanjing Qinglongshan Animal Center, and the research met the guidelines of their animal ethics committee and was approved by the ethics committee. The research was approved by Ethics Statements 2020≈gl18 from the Laboratory Animal Ethics Committee of the Drum Tower Hospital, Nanjing University School of Medicine. For the clinical trial, ethics approval was received from the Medical Ethics Committee of the Drum Tower Hospital, Nanjing University (reference number: 2023‐435‐02).

## Conflict of Interest

The authors declare no conflict of interest.

## Author Contributions

Z.L. and Y.D. contributed equally to this work and co‐first authors. Z.L. performed conceptualization, data curation, formal analysis, methodology, validation, visualization, wrote the original draft, and reviewed and edited the final manuscript. Y.D. performed data curation, methodology, software, visualization, wrote the original draft, and reviewed and edited the final manuscript. T.Z. performed visualization and software. T.Q. performed visualization, wrote the original draft, and reviewed and edited the final manuscript. Y.Y. performed Validation. W.X. performed Investigation. M.F. contributed to investigation, methodology, and software. B.C. performed funding acquisition and supervision. X.W. performed funding acquisition, supervision, wrote the original draft, and reviewed and edited the final manuscript. P.X. performed funding acquisition, project administration, resources, supervision, wrote the original draft, and reviewed and edited the final manuscript.

## Supporting information

Supporting Information

## Data Availability

The data that support the findings of this study are available from the corresponding author upon reasonable request.
