# Supplementary material for: A Novel Engineering Cell Therapy Platform Mimicking the Immune Thrombocytopenia‐Derived Platelets to Inhibit Cytokine Storm in Hemophagocytic Lymphohistiocytosis
Source: Adv Sci (Weinh). 2024 Sep 11;11(45):2404571. doi: 10.1002/advs.202404571 (PMC11615807; doi:10.1002/advs.202404571)
Supplement: Supplementary file 1 — Supporting Information [file ADVS-11-2404571-s001.pdf]

ADVANCED SCIENCE
Open Access

Supporting Information

for *Adv. Sci.*, DOI 10.1002/advs.202404571

A Novel Engineering Cell Therapy Platform Mimicking the Immune Thrombocytopenia-Derived Platelets to Inhibit Cytokine Storm in Hemophagocytic Lymphohistiocytosis

Zhenyu Liu, Ying Du, Tong Zhou, Ting Qin, Yining Yuan, Weilu Xu, MengKun Fang, Xuemei Wang, Bing Chen* and Peipei Xu**

A Novel Engineering Cell Therapy Platform Mimicking the Immune Thrombocytopenia-derived Platelets to Inhibit Cytokine Storm in Hemophagocytic Lymphohistiocytosis

Zhenyu Liu[1]#, Ying Du[1]#, Tong Zhou[1], Ting Qin[2], Yining Yuan[1], Weilu Xu[1], MengKun Fang[1], Xuemei Wang*[3], Bing Chen*[1], Peipei Xu*[1]

[1]Department of Hematology, Nanjing Drum Tower Hospital, Affiliated Hospital of Medical School, Nanjing University, Nanjing 210008, China

[2]Department of Hematology, Nanjing Drum Tower Hospital Clinical College of Nanjing Medical University

[3]School of Biological Science & Medical Engineering, Southeast University, Nanjing 210096, China

*Correspondences: xupeipei0618@nju.edu.cn (Peipei Xu); chenb211@163.com (Bing Chen); 101005183@seu.edu.cn (Xuemei Wang)

321 Zhongshan Road, Nanjing 210008, Jiangsu Province

Supplementary Figures

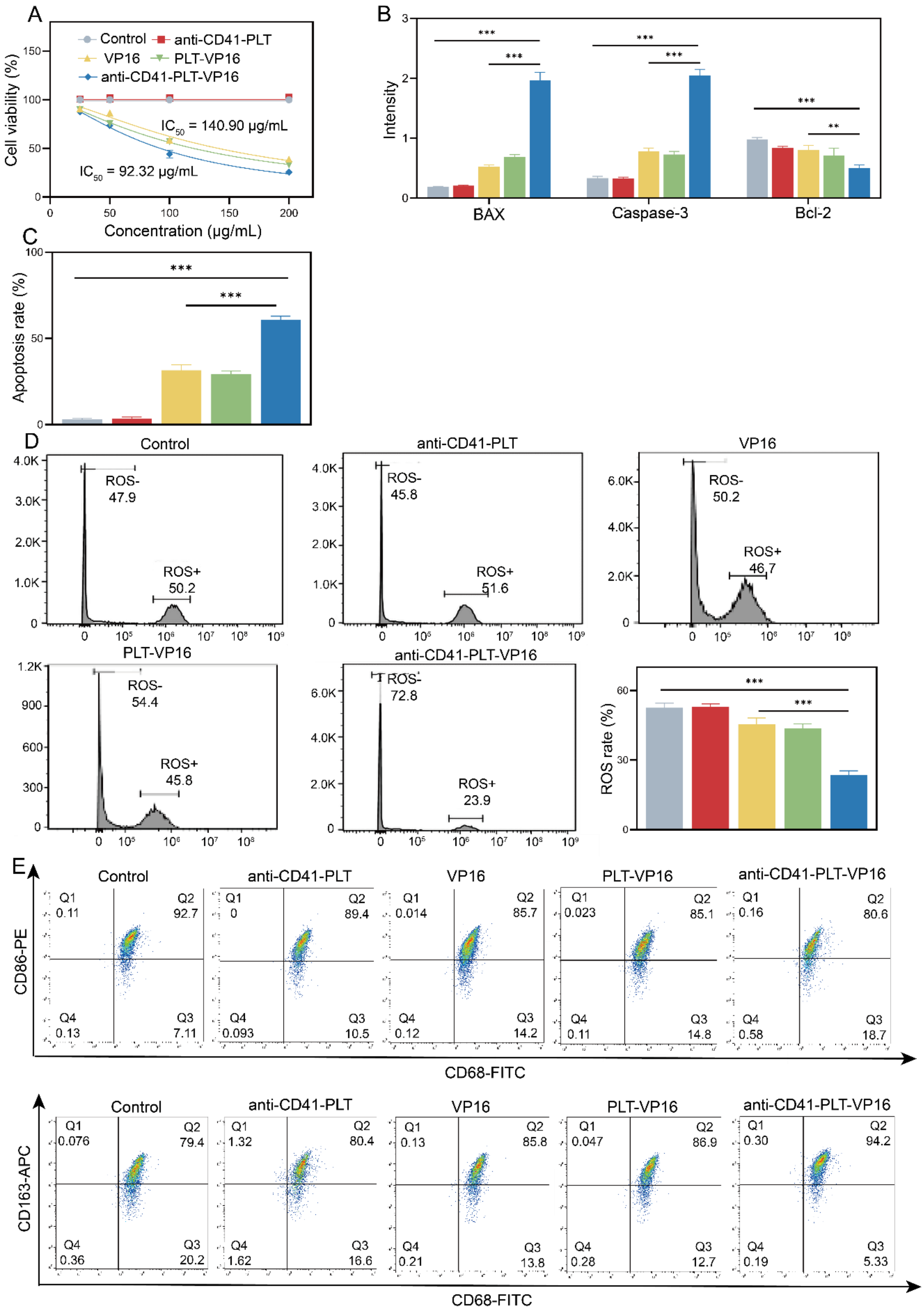

Figure S1. Characterization of anti-CD41-PLT-VP16 and its effect on macrophages. (A) IC50 in the CCK-8 assay. The IC50 of the VP16 monotherapy group was 140.90 μg/mL, while the IC50 of the anti-CD41-PLT-VP16 group was 92.32 μg/mL. (B) The expression of Bax, Caspase-3, and Bcl-2 in the mitochondrial apoptosis pathway was detected by WB analysis. (C) Annexin V-FITC/PI was used to detect the 12 h apoptosis rate of macrophages in the different treatment groups. (D) FCM detection of ROS levels in macrophages from different treatment groups. (E) FCM detection of macrophage polarization. $CD68^{+}CD86^{+}$ cells were defined as M1, while $CD68^{+}CD163^{+}$ cells were defined as M2. $^{**}P < 0.01$, $^{***}P < 0.001$.

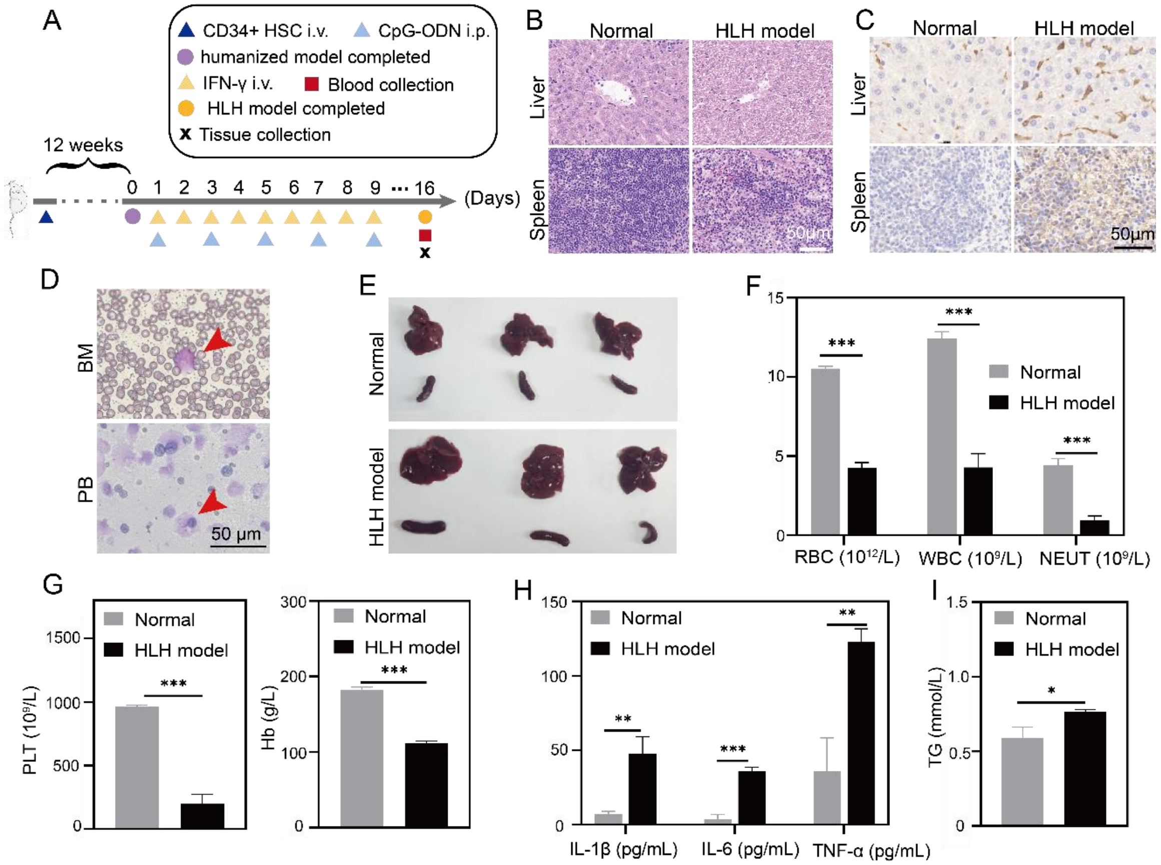

Figure S2. Successful construction of the HLH mouse model. (**A**) Schematic diagram of the humanized mouse model. (**B**) H&E staining of liver and spleen tissues from the HLH model group and control group (n = 6). (**C**) IHC staining of F4/80+ in liver and spleen tissues from HLH model and control mice (n = 6). (**D**) Riesling staining of BM and PB smears of HLH model mice with macrophage hemophilia shown by arrows (n = 6). (**E**) Photographs of liver and spleen sizes in the HLH model group and control group (n = 6). (**F&G**) Blood parameters, including RBC, WBC, PLT count, neutrophil count, and Hb level (n = 6), of mice in the HLH model group and control group. (H) Cytokine levels, including those of IL-1β, IL-6, and TNF-α, in the PB of HLH model mice and control mice (n = 6). (I) TG levels in HLH model mice and control mice (n = 6). *P < 0.05, **P < 0.01, ***P < 0.001.

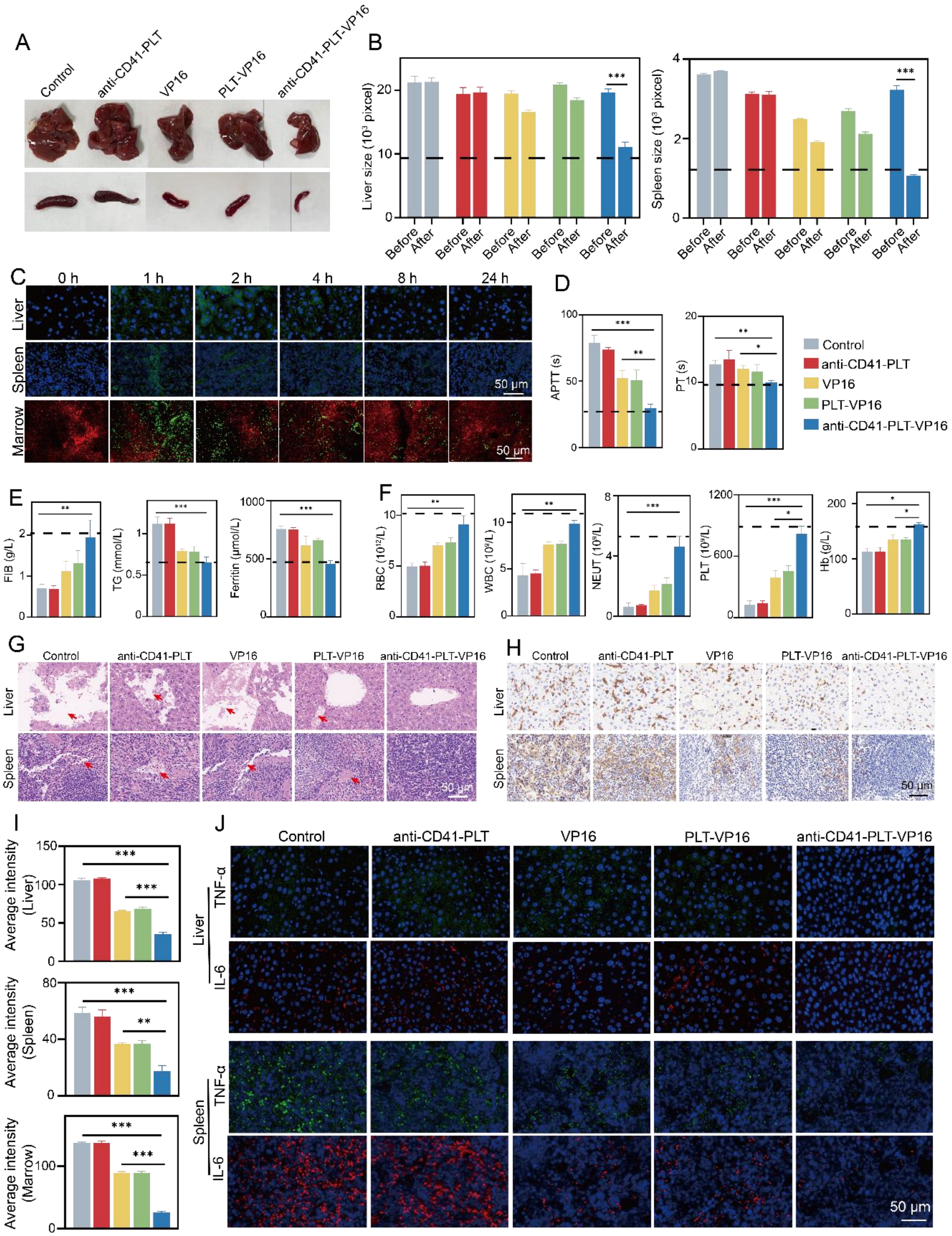

Figure S3. Therapeutic effects of different treatments on mice with HLH. (**A**) Photographs of changes in the size of liver and spleen tissues of mice in different treatment groups, from left to right, NS, PLT, VP16, PLT-VP16, and anti-CD41-PLT-VP16 groups (n = 6). (**B**) Quantitative analysis of liver and spleen MR images of mice before and after treatment in each group (n = 6).

Liver and spleen enlargement in mice in the anti-CD41-PLT-VP16 group was significantly alleviated. The dashed line represents the liver and spleen volume of normal humanized mice. Dashed lines indicate normal values. (**C**) Changes in the fluorescence signal intensity in mouse liver and spleen tissues after FITC-conjugated anti-CD41-PLT-VP16 was injected through the tail vein (n = 6) (DAPI: blue, FITC: green; scale bar = 50 μm). Changes in FITC fluorescence over time in the BM of mice after injection of FITC-conjugated anti-CD41-PLT-VP16 through the tail vein were photographed by IVM (n = 6). (Blood: red, FITC: green, bar = 50 μm.) (**D**) Coagulation profile of mice in different treatment groups, including APTT and PT (n = 6). Dashed lines indicate normal values. (**E**) FIB, TG, and ferritin levels in mice in different treatment groups (n = 6). Dashed lines indicate normal values. (**F**) RBC, WBC, PLT, Hb, and neutrophil levels in mice in different treatment groups (n = 6). Dashed lines indicate normal values. (**G**) H&E staining of liver and spleen tissues from mice in different treatment groups (n = 6). (**H**) IHC staining demonstrating the levels of F4/80+ in the liver and spleen of mice from different treatment groups (n = 6). (**I**) Results of quantitative analysis of IVM imaging in mouse liver, spleen and BM. (**J**) Immunofluorescence assays demonstrated the levels of TNF-α and IL-6 in the liver and spleen of mice from different treatment groups (n = 6). (DAPI: blue, TNF-α marker: green, IL-6 marker: red, bar = 50 μm) *$P < 0.05$, **$P < 0.01$, ***$P < 0.001$.

Table S1. The baseline characteristics of the assessed HLH patients

Characteristics	Trail group (n = 9)	Control group (n = 15)
Gender	Case n (%)	
Female	2 (22.2)	4 (26.7)
Male	7 (77.8)	11 (73.3)
Age [Mean (range)]	39 (23-57)	35 (19-62)
Patients meeting HLH criteria at baseline		
Fever	9 (100.0)	14 (93.3)
Splenomegaly	6 (66.7)	12 (80.0)
Thrombocytopenia	8 (88.9)	10 (66.7)
Neutropenia	9 (100.0)	15 (100.0)
Hyperferritinemia	9 (100.0)	14 (93.3)
Hypofibrinogenemia	9 (100.0)	15 (100.0)
Hypertriglyceridemia	4 (44.4)	9 (60.0)
Reduced NK activity	7 (77.8)	11 (73.3)
sCD25 (sIL-2r) ≥ 2400 U/mL	9 (100)	14 (93.3)
Patients with other HLH abnormalities at baseline		
D-dimers ＞ 500 μg/L	2 (22.2)	4 (26.7)
ALT > 125 IU/L	9 (100)	14 (93.3)
LDH >1000 IU/L	3 (33.3)	6 (40.0)
CNS symptoms	1 (11.1)	3 (20.0)

Table S2. Outcomes for previous HLH clinical trial

Characteristics	Trail group (n = 9)	Control group (n = 15)
Fever	0 (0)	10 (66.7)
Splenomegaly	2 (22.2)	11 (73.3)
Thrombocytopenia	1 (11.1)	6 (40.0)
Neutropenia	1 (11.1)	11 (73.3)
Hyperferritinemia	2 (22.2)	8 (53.3)
Hypofibrinogenemia	0 (0)	9 (60.0)
Hypertriglyceridemia	3 (33.3)	9 (60.0)
Reduced NK activity	0 (0)	7 (46.7)
sCD25 (sIL-2r) ≥ 2400 U/mL	1 (11.1)	11 (73.3)
D-dimers ＞ 500 μg/L	1 (11.1)	5 (33.3)
ALT > 125 IU/L	3 (33.3)	12 (80.0)
LDH >1000 IU/L	0 (0)	2 (13.3)
CNS symptoms	0 (0)	2 (13.3)
Outcomes		
Complete response	6 (66.7)	3 (20.0)
Partial response	2 (22.2)	2 (13.3)
No response	1 (11.1)	10 (66.7)
